# Distance sampling of duikers in the rainforest: Dealing with transect avoidance

**DOI:** 10.1371/journal.pone.0240049

**Published:** 2020-10-08

**Authors:** Gaïus Elenga, Christophe Bonenfant, Guillaume Péron

**Affiliations:** 1 Laboratoire de Biométrie et Biologie Évolutive UMR5558, Univ Lyon, Université Lyon 1, CNRS, Villeurbanne, France; 2 Department of the Environment, Faculty of Sciences, University of Kinshasa, Kinshasa, Democratic Republic of Congo; University of Bucharest, ROMANIA

## Abstract

Bushmeat is a major source of protein and income in tropical regions but is often over-harvested. A better monitoring of bushmeat stocks could help achieve sustainability. We used a combination of simulations and transect survey data collected from blue duikers (*Philantomba monticola*) in the Lomako wildlife reserve, Democratic Republic of the Congo, to investigate the use of transect-based distance sampling to monitor bushmeat stocks. The comparison of dung piles and direct observations of duikers evidenced that animals avoided both the transects in the absence of observers, and the observers themselves. This type of behavioural response appeared common in a literature survey. It causes a negative bias in the estimates of population densities from the standard distance sampling methodology. This negative bias would lead to over-pessimistic predictions of population viability, especially if the behavioural response is more intense in the locations where the animals are hunted. In turn, this would lead to excessively conservative management recommendations. To correct for the effect of the behavioural response of the animals to either the transects or the observers, we recommend recording both the forward and perpendicular distances to the observers (2D distance sampling), not just the perpendicular distance. We also recommend multiple-observer protocols. As a cautionary note, we also demonstrate a scenario where the intensity of the behavioural response is too high to reliably estimate the abundance of the population. As a perspective, we outline the general principles of a local stakeholder-based program combining distance sampling with less intensive types of ecological indicators to monitor wildlife populations.

## Introduction

Over-harvesting jeopardizes the food safety, livelihood, and cultural identity of people who consume bushmeat, i.e., meat from wild mammals, birds, and reptiles, typically in tropical, forested environments [1–4]. Local extinctions are regularly reported in exploited forests, especially for large animal species [5–8]. Management recommendations such as hunting quotas are typically based on an estimate of the optimal sustainable yield, itself coming from an estimate of the population abundance of the harvested species [5, 9, 10]. Recently, less data-hungry approaches have been advocated that are based on “Indicators of ecological change” [11]. These indicators of sustainability take into account the natural balance between populations and their environment, for example, density-dependence under a stochastic regime of variable food abundance [12, 13]. They focus on variation in body condition, in primary production, in the intensity of herbivory, and in the relative abundance of different species in a guild [11, 14, 15]. They are typically much easier to track than the population abundance. However to validate these indicators in a new system, we still need pilot data about how the population abundance changes over time and under different harvest rates. Hereafter we focus on such a pilot study, acknowledging that the objective for wildlife managers is to ultimately apply less cumbersome monitoring protocols [11].

The main objective is to account for the imperfect detection of individuals during surveys in the field, *i*.*e*. the fact that a fraction of the animals is missed by the observers during the surveys [16]. That fraction can be quite variable over time, across different sites, observers, survey methodologies, species, and age or sex classes within species [17, 18]. Thereby, when comparing the number of detected individuals over time for example, the inference can be flawed by temporal variation in detection probability. This is called a statistical bias or detection bias: the metric (the number of detected individuals) consistently deviates from the target variable (the population abundance). Here the bias would be consistently negative (fewer individuals are detected than were present) but of an unknown and variable magnitude (we do not know how many were missed and it could vary from one count to the next). The most widely considered method to correct for that detection bias is transect-based distance sampling [19, 20]. In distance sampling, the first step is to estimate the shape of the “detection function” that represents how the probability to detect an individual decreases with the distance from the observer [21]. The second step is to compute the average detection probability over the survey area and use that to correct the raw count and obtain the unbiased population density of the target species [19].

**Fig 1:**
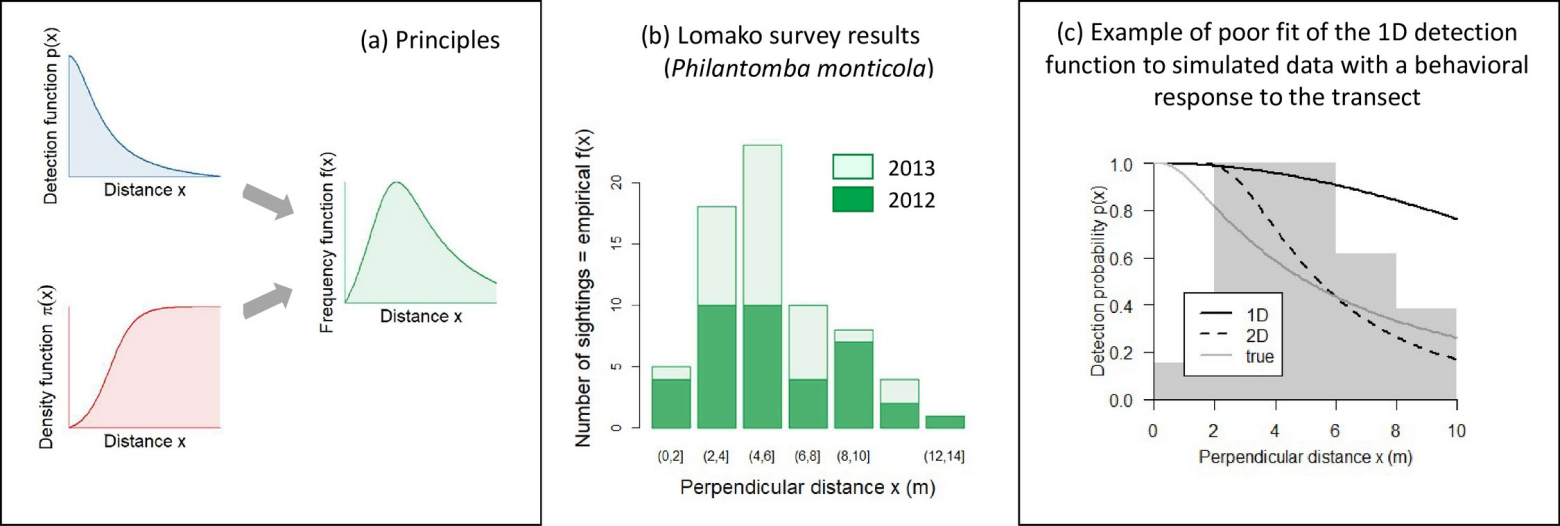
When the animals avoid the transect or the observer, there can be a peak in sightings at intermediate distances (panel a: theoretical principles; panel b: empirical observation). This pattern causes a lack of fit of the standard, 1D distance sampling detection function (panel c; the grey histogram is the density of sightings), because that function wrongly assumes a detection rate of 1 on the transect and a monotonous decline in the frequency of sightings with the distance to the transect.

Applying distance sampling in forested environments remains challenging because of the low visibility, meaning that the rate at which detection probability decline with distance is much faster than in open environments. In addition, the animals may avoid the transects from which they are surveyed. They may respond to the change in vegetation around the transects, meaning that they always avoid the transect even in the absence of observers. Or they may respond to the presence of an observer, who they usually perceive as a threat especially in hunted areas [22–25]. They may respond by moving away from the threat or by changing their behaviour to become less detectable [26–28]. These different types of avoidance behaviour seem to be widespread among forest-dwelling mammals (Table 1), and perhaps especially so when the transects are used not only for scientific purposes, but also for hunting [29, 30]. They all have the same consequence statistically speaking [12, 24, 29]. When any of these behaviours occurs, the number of detections does not decrease steadily with distance from the transects, as is assumed to be the case in standard distance sampling. Instead the number of detections exhibits a peak at intermediate distances [22–25] (Fig 1). We hereafter use the phrase “behavioural response” to refer to this situation in general.

**Table 1:** A non-exhaustive review of studies evidencing a behavioural response to the transects in forest- or woodland-dwelling mammals. We only compiled positive reports of behavioural responses. “Distance histogram” means that the distribution of recorded distances followed a shape like in Fig 1, sometimes with additional evidence from further modelling. “GPS tracking” means that geo-tracked individuals avoided the vicinity of the transects more than expected by chance. “Camera trapping” means that the frequency of pictures decreased near transects. “Counts” means that population abundance was larger in a block without transect than in a block with transect.

Species	Location	Nature of the evidence	Reference
**Ungulates**			
*Cephalophus natalensis harveyi*	Udzungwa Mountains, Tanzania	Distance histogram	[37]
*Cephalophus ogilbyi*, *Cephalophus jentinki*, *Cephalophus sylvicultor* and *Cephalophus zebra*	Taï National Park, Côte d’Ivoire	Counts	[63]
*Capreolus capreolus*	North Yorkshire, UK	Distance histogram	[65]
*Capreolus capreolus*	Central Germany	Distance histogram	[66]
*Dama dama*	Castelporziano, Italy	GPS tracking	[67]
*Odocoileus virginianus*	Catoctin and Antietam NP, USA	Camera trapping	[68]
*Odocoileus virginianus*	Gettysburg Park, USA	GPS tracking	[69]
*Odocoileus virginianus*	Blue Hills Reservation, USA	Distance histogram	[70]
*Sus scrofa*	Castelporziano, Italy	GPS tracking	[67]
*Cervus nippon*	Nikko National Park, Japan	Distance histogram	[71]
*Cervus nippon*	Yakushima Island, Japan	Distance histogram	[72]
*Cervus elaphus*	Kaupanger, Norway	Camera trapping	[73]
**Primates**			
*Nycticebus bengalensis*	Khao Ang Rue Nai Wildlife Sanctuary, Thailand	Distance histogram	[74]
*Callithrix penicillata*	South-east Brazil	Counts	[75]
*Procolobus gordonorum*	Udzungwa Mountains, Tanzania	Distance histogram	[76]
*Colobus satanas*	Makandé, Gabon	Distance histogram	[58]
*Colobus angolensis*	Udzungwa Mountains, Tanzania	Distance histogram	[76]
*Cercopithecus mitis*	Udzungwa Mountains, Tanzania	Distance histogram	[76]
*Gorilla gorilla*	Dja Biosphere Reserve, Cameroon	Counts	[30]
*Pan troglodytes*	Dja Biosphere Reserve, Cameroon	Counts	[30]
*Pan paniscus*	Salonga, DRC	Counts	[77]
**Others**			
*Macropus giganteus* and *Macropus rufus*	Queensland, Australia	Distance histogram	[24]
*Macropus giganteus*	Canberra, Australia	GPS tracking	[22]
*Cuniculus paca*	Ilha Grande, Brazil	Distance histogram	[78]
*Dasyprocta prymnolopha*	Belo Horizonte, Brazil	Counts	[79]
*Vulpes vulpes*	France	Distance histogram	[80]
*Ursus arctos*	Alaska (3 parks)	Distance histogram	[81]

In the presence of a behavioural response, fitting one of the usual formulae for the distance sampling detection function would lead to a major underestimation of the population abundance, because the function is ill-fitted to the situation (Fig 1) [23]. The magnitude of the bias could depend on the intensity of the behavioural response. For instance if the behavioural response increases with the hunting pressure [31], the population abundance would be more severely under-estimated by distance sampling in hunted than in non-hunted areas. This mechanism might be one of the reasons why the optimal yield projections based on transect data have repeatedly proven unreliable [10]. In other words, researchers, because they rely on artificially low estimates of population density and artificially high estimates of the effect of hunting on population density, have apparently been generating over-pessimistic estimates of the population viability of bushmeat species. For example, the sustainable harvest rate for duikers (*Cephalophus* and *Monticola* sp.) varies by an order of magnitude depending on the survey method used to estimate it (Table 4 in [10]). Tapirs *Tapirus terrestris* have been shown to persist in areas where they should theoretically go extinct within years [32] (but in the case of tapirs the explanation appears to be biological, not methodological [32]).

**Fig 2:**
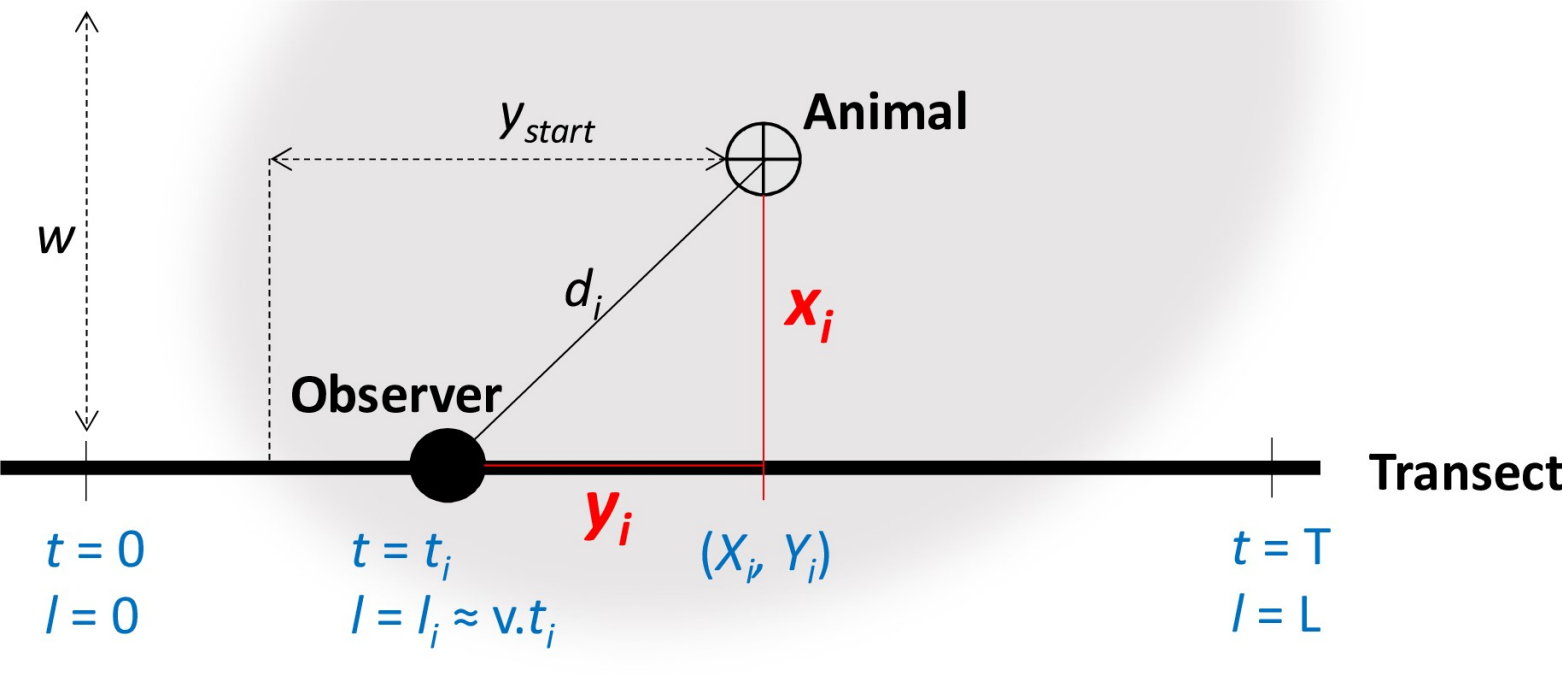
Principles of the 2D distance sampling framework [33]. The observer is denoted by a filled circle that progresses along a transect, taking position *l*_*i*_ at time *t*_*i*_. The animal is denoted by a crossed circle, surrounded by the “detection kernel” in grey. The distance to the observer is recorded in two dimensions, with *y*_*i*_ the forward distance and *x*_*i*_ the perpendicular distance. In the standard, 1D framework, only *x*_*i*_ is recorded. If the observer moves at a constant speed *v* along the transect (e.g., survey from a vehicle or boat), then *y*_*i*_ can be inferred from the geographical coordinates (*X*i,*Y*_*i*_) and the time at the detection *t*_*i*_. The transect half-width *w* is the cut-off perpendicular distance beyond which animals are ignored, typically defined *a posteriori* based on the maximum recorded distance. The effective forward distance *y*_*start*_ is roughly the maximum forward distance at which an animal that is on the transect can be recorded.

Borchers and Cox [33] recently brought to the fore the “2D version” of transect-based distance sampling. “2D distance sampling” involves recording both the perpendicular distance from the transect, and the forward distance parallel to the transect (Fig 2). By contrast in standard distance sampling only the perpendicular distance is recorded [19]. In this study we bring some more light on the 2D distance sampling methodology, and we highlight its relevance in the presence of a behavioural response to the transects and in the specific context of bushmeat stock assessment in the rainforest. The 2D framework makes it possible to fit more complex distance functions that separate the behavioural response from the effect of distance on detection probability. By doing so, we remove the aforementioned negative bias on the population abundance estimate [23, 33, 34]. Recording the forward distance in addition to the perpendicular distance involves an only minor change in the distance sampling protocol. Since the distance sampling protocol has already been successfully applied in the rainforest environment [17, 18, 35], 2D distance sampling appears to represent an adequate solution to monitor bushmeat stocks when the bushmeat species exhibit a behavioural response to the transects.

Our study is motivated by surveys of blue duiker (*Philantomba monticola)* in the Democratic Republic of the Congo. Duikers are an African lineage of small to medium-sized forest antelopes that represent a major source of bushmeat to many people throughout their distribution [36]. We distinguished between the response of duikers to the transects themselves, without observers, and the response of duikers to the observers. To do that, we compared the distribution of dung piles and the distribution of direct observations. The distribution of dung piles according to the distance to the transects should quantify the response to the transects themselves, without observers. The difference between dung piles and direct observations should document the response to the observers. We expected that the response to the observers would be larger than the response to the transects alone. We then used simulations to quantify the effect of these behavioural responses on population density estimates. These simulation results apply to any species that exhibit a behavioural response to the transects or to the observers, not just blue duiker. Lastly, we computed the ratio between the estimated number of dung piles and the estimated number of live animals. This ratio is important in practice because dung piles are easier to record than live animals; but converting the density of dung piles into a density of animals requires a precise estimate of the local conversion ratio (see Discussion). We investigated whether the behavioural response affected the estimate of the conversion ratio.

Lastly, we also aimed to remind researchers of the eventuality that the behavioural response can be too intense to estimate population abundance, i.e., there is a limit to the amount of imperfections that statistical routine can correct. The detection rate can be so low that the data does not contain enough information about the abundance away from the transects [37]. This demonstration is included in the simulation section.

## Material and methods

The field protocol was approved by the Ministry of Environment and Sustainable Development of the DRC and by the Institut Congolais pour la Conservation de la Nature. The study does not involve physical captures.

### The 2D distance sampling framework

In the early distance sampling literature, researchers recorded the position of the animals in a 2D referential [34]. In transect-based distance sampling, this means recording both the forward distance *y*, which is the distance of the observer to the nearest point to the animal on the transect, and the perpendicular distance *x*, which is the distance from that point on the transect to the animal (Fig 2). The simplified protocol currently in widespread use instead requires only the perpendicular distance *x* [19]. One of the reasons why the distance sampling protocol was simplified and researchers stopped recording the forward distance is that Hayes & Buckland [34] recognized that “the validation of any particular model using field data would be difficult” and that “right-angle distances may be difficult to measure accurately (…) when the radial distance is large but the right-angle distance is small”. Therefore, under the assumption that the detection probability does not depend on the angle of observation, it was much simpler to use only the perpendicular distance.

Following Borchers and Cox [33], we revive the analysis of 2D distance data. In practice, we customized the functions from the R-package LT2D [33] (to simplify the interface, resolve a few typos, and make the framework amenable to extensive simulations). We modelled the behavioural response of animals as a sigmoid function, *i*.*e*. duiker abundance gradually increased with distance from the transect (Fig 1A). Importantly, this implies that the animals always avoid the observers when they are at distance zero (but they may quickly resume normal abundance and availability to detection as soon as their distance is non-zero). We modelled the detection function in two dimensions using a radial exponential hazard risk (*h*_HB_ under the notation of Borchers & Cox [33]), thereby making the same approximation as the half-normal detection function that is commonly found to describe the detection process in 1D [21]. Importantly, this implies that observers detect all the animals that are at perpendicular distance zero (but the detection probability may rapidly decline for non-zero distances, and in addition because of the sigmoid model above the animals are never at distance zero).

In the above-described particular parameterization of the 2D distance sampling model, it is not possible to record a perpendicular distance of zero (Fig 1). This may require some manipulation of the data to change zeroes into a small non-zero value. Alternatively, and importantly, we also provide alternative models in the code appendix (S1 Appendix). These alternative models can accommodate the violation of the above assumptions. For example, if the detection probability at distance zero is already imperfect, use detection kernel “h.yTRE” instead of “h.RE” as currently implemented. If the animals do not completely avoid the transect even at distance zero from the observer, use behavioural response function “pi.sigmoI” (for “sigmoid with Intercept”) instead of “pi.sigmo” as currently implemented. Note that these two functions h.yTRE and pi.sigmoI are incompatible with each other (cannot be used simultaneously in the same model), and that they feature one more parameter than the functions they replace.

The parameters of the two functions (the sigmoid behavioural response function and the detection function with radial exponential detection hazard) were simultaneously estimated by fitting the model to the recorded distance data using a maximum-likelihood approach. We hereafter use the established phrases “effective strip half-width” for the average detection distance should the animals not exhibit any behavioural response, and “average detection probability” for the average probability of being both available for detection and detected. The average detection probability therefore combines the behavioural response and the detection probability *stricto sensu*. To compare our results to those from the 1D framework, we used the Distance package for R [38], more precisely, the ‘ds’ function with default options.

### Duiker data collection

We surveyed duikers from 5 transects totalling 31km in the Lomako Yokokala Wildlife Reserve (0° 56′ 00″ N, 21° 20′ 00″ E). The five transects were parallel and separated by 1 km. We walked each transect twice in March-April 2012 and twice in November 2013, performing one of the surveys by day for dung piles and the other survey by night with a powerful spotlight for direct observations. We followed the established distance sampling protocol, i.e., we did not record the forward distance. Upon detection of a duiker or dung pile, we immediately walked to the nearest point on the transect, and recorded the time, GPS coordinates, and perpendicular distance of the initial location of the duiker, or of the dung pile, to the transect. When sampling dung piles, we ignored the piles that were considered older than a day old by experienced trackers upon visual examination. A preliminary investigation of the recorded perpendicular distance distribution suggested that the blue duikers avoided the transects or the observers or both (Fig 1B).

### Lomako data simulation

**Fig 3:**
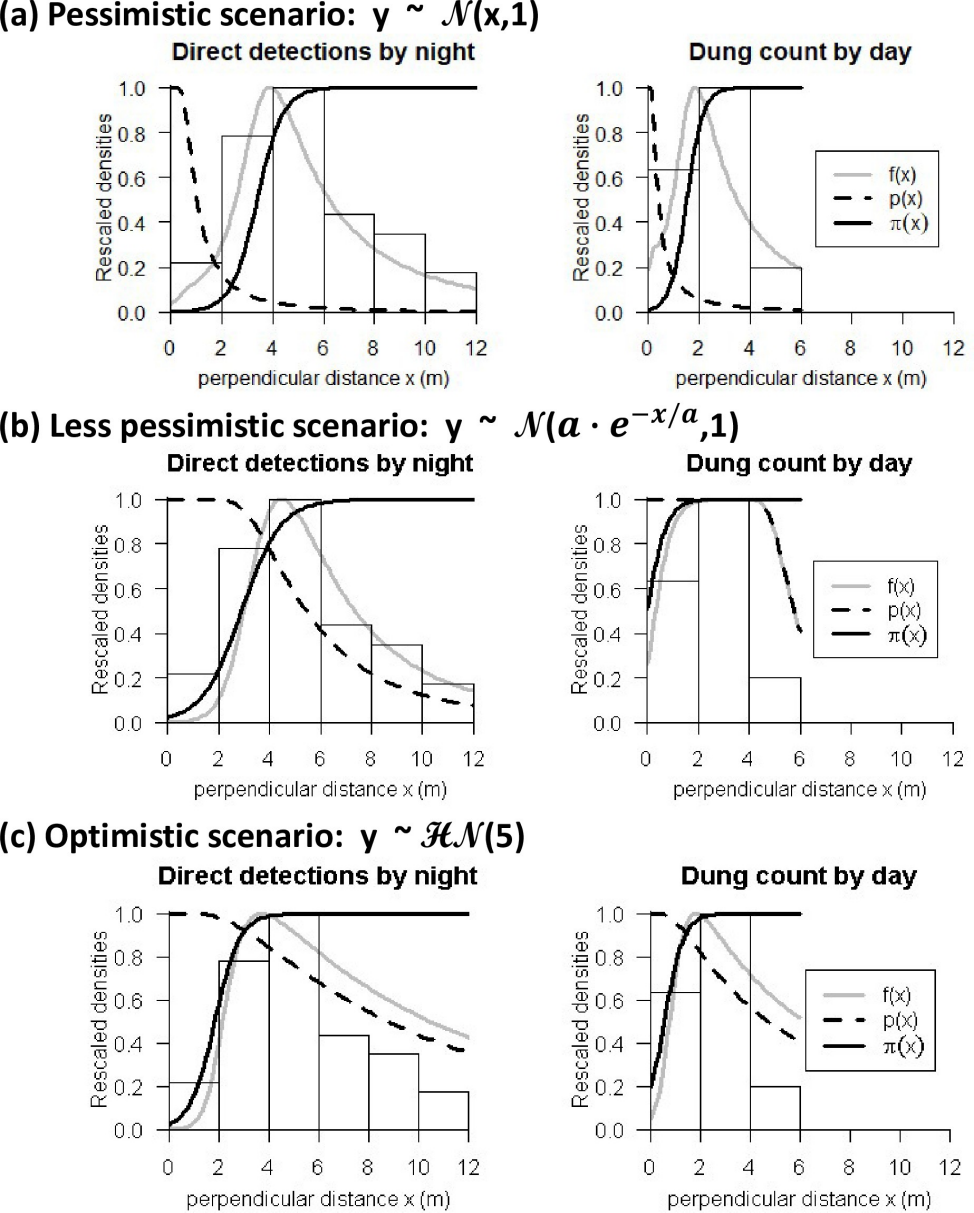
Estimated detection probability *p*(x), behavioural response π(x), and expected density of sightings *f*(x), as a function of the perpendicular distance *x*, for blue duikers in the Lomako study area, with three ways to simulate the forward distance y. The binned detection data are provided for comparison (histogram).

Because we did not record the forward distances in the field, and in order to illustrate our points about 2D distance sampling, we generated artificial forward distances associated to each Lomako record, following three scenarios (Fig 3; simulation code in S1 Appendix):

- A pessimistic but likely scenario. We simulated the forward distances to be on average the same as the observed perpendicular distances. We added to the recorded perpendicular distance a random Gaussian noise with a mean of 0 and a standard deviation of 1 meter in order to generate some variability between replicates of the simulation. Any negative distance value resulting from an excessive negative noise we reset to zero meter.
- A somewhat less pessimistic scenario. In this scenario there is a negative correlation between the forward and perpendicular distances. In other words, when the duiker is on the transect, it is easier to detect and is spotted from a greater forward distance than when the duiker is further from the transect. We used an inverse exponential relationship (Fig 3) with the half distance parameter *a* set to half the maximum recorded perpendicular distance. We added onto that expected distance a random Gaussian noise with a mean of zero and a standard deviation of 1 meter. Any negative distance value resulting from an excessive negative noise we reset to zero meter.
- An optimistic (but unlikely) scenario. The forward distances were relatively large in this scenario, meaning easy detection, and they were independent from the perpendicular distance. We simulated this scenario using a half-normal distribution function with half-detection distance set to 5 meters.

### Artificial data simulation

We simulated 150 completely artificial datasets to quantify the bias in population density estimates induced by the behavioural response. We generated the datasets according to the above-described sigmoid-radial exponential model. Our simulations featured 100 animals, which is the order of magnitude of the surveyed population sizes in many real-life applications. We considered 4 scenarios with low or high detectability, and strong or weak behavioural response to the transect. More precisely, the *b* vector that controls the shape of the detection function (notation as in [33]) could be [0.25,0.25] or [1.75, 1.75]. The log(φ) vector that controls the location and aspect of the sigmoid behavioural response function could be [−4, −1] or [−4, −6]. We applied the standard 1D distance sampling and the 2D distance sampling routines to the 150 datasets. We computed the bias of the estimates relative to the known simulated abundance, which quantifies the presence and magnitude of a systematic error in the estimate of population abundance, and the root-mean squared error (RMSE), which measures the variability of the error across simulations and therefore the expected precision of the estimate of population abundance.

## Results

### Bias in abundance estimates

In Lomako, there was almost no direct detection less than 2 meters from the transect and a peak in sightings occurred at 5 meters from the transect (Fig 1B). These patterns are exactly those we expect when the animals avoid the transects or the observers (Fig 1A). Using the 1D distance sampling methodology, we estimated a density of 119 blue duikers per km² (95% CI: 53–180) on average over the two years. The ratio of dung piles per live animal estimated from the 1D distance sampling framework was 1.8 (95% CI: 0.7–2.7).

In all three scenarios, the fitted model indicated a behavioural response both in the dung pile data and in the direct observation data (Fig 3). The behavioural response was consistently more intense in the direct observation data than in the dung data (Fig 3: bold black lines). Therefore, the animals responded both to the transects and to the observers. The response to the observers was almost an order of magnitude stronger than the response to the transects alone (Fig 3).

In the artificial datasets that did not use the Lomako data, the 1D framework over-estimated the detection rate (for example: Fig 1C, solid black line vs. other lines). This over-estimation of the detection rate propagated into an under-estimation of the abundance by 20 to 70% (Table 2: “bias 1D”). Applying the 2D method to the same simulated datasets solved the problem for the most part (Table 2: “bias 2D”). However, some bias remained in the most challenging scenario that combined low detectability and strong behavioural response (Table 2). This is because in this scenario the sample size of recorded detections was small compared to other scenarios, especially at short distances which are the ones the model uses to estimate the behavioural response. In addition, when detection probability was high, the 2D framework tended to slightly over-estimate abundance (Table 2). This is likely because, even if the simulating model was a sigmoid-radial exponential model like the fitted model, some idiosyncrasies in the realized distance distribution caused a lack of fit of the sigmoid-radial exponential model to the simulated data. Lastly, because the 2D model has more parameters than the 1D model, the precision of the estimates was often lower with the 2D than with the 1D model (Table 2: “RMSE 1D” vs. “RMSE 2D”), as expected from the bias-precision trade-off.

**Table 2:** Quantifying the bias and Root Mean Squared Error (RMSE) of the abundance estimated using 1D (where only the perpendicular distance to transect is recorded) and 2D (where both the perpendicular and forward distances are recorded) distance sampling, in four scenarios of detectability and intensity of the behavioural response to the transect. Low detectability means that the detection probability declines quickly with distance to the transect. Strong behavioural response means that the increase in animal abundance with distance from the transect is slow, meaning that they rarely occur near the transect. Results are given in %. A bias of -50% means that the abundance was under-estimated by 50% on average over 150 replicates.

Detectability	Behavioural response	Bias 1D	Bias 2D	RMSE 1D	RMSE 2D
High	Weak	-21	10	23	26
High	Strong	-39	7	39	47
Low	Weak	-33	-19	35	29
Low	Strong	-71	-34	71	72

### Sometimes the animals are just too shy

In the pessimistic scenario, the estimated behavioural response to the transect was very strong (Fig 3A, bold black line). The estimated distance at which duikers started to be abundant was beyond twice the effective strip half-width, which means that almost all the duikers remained out of view. The estimated average detection probability was below 5% (95% CI: 0–100). Therefore, the study system under the pessimistic scenario was too challenging to apply distance sampling, and the 2D results made that situation obvious.

In the less pessimistic scenario, the estimated behavioural response to the transect was less marked than in the more pessimistic scenario (Fig 3B, bold black line). However, there was evidence of lack of fit in the form of a lack of predicted detections in the 2–4 meter bin (Fig 3B, histogram vs. grey line). The estimated average detection probability was 34% (95% CI: 20–49%), the abundance estimate was 251 duikers per km² (95% CI: 178–458), of which 29% were estimated to be unavailable because of the behavioural response to the transect, and the ratio of dung piles per live animal was 0.6 (95% CI: 0.4–0.9).

In the optimistic scenario, the estimated detection probability was unrealistically high (e.g., 40% detection rate at 10 m from the transects; Fig 3C, dashed line). The model exhibited lack of fit in the form of an excess of predicted detections in the furthest distance bins (Fig 3C, histogram vs. grey line). The estimated average detection probability was 61% (95% CI: 43–74%), the abundance estimate was 138 duikers per km² (95% CI: 114–212), of which 30% were estimated to be unavailable because of the behavioural response to the transect, and the ratio of dung piles per live animal was 1.6 (95% CI: 1.5–1.6).

## Discussion

Based on the distribution of dung piles, blue duikers appeared to permanently avoid the 2-meter strip on each side of the transects in the forest. Their response when an observer was present, although stronger than their response to the transect alone, appeared to also wane after just 5 meters (Fig 3: direct detections by night). Nevertheless, this response was sufficient to have a major effect on the estimates of population abundance (Table 2) [22–25, 33]. This is because the probability of detection declined rapidly within mere meters of the transect (Fig 2), meaning that the behavioural response, although small, was significant in comparison to the effective strip width.

Our report that duikers responded behaviourally to both the transects and the observers is congruent with other studies on duikers [37, 39]. The literature (Table 1) suggests that these patterns are common among other forest- and savannah-dwelling mammals, including carnivores, large herbivores, and primates. The behavioural response is actually probably even stronger in environments with more visibility than our study area [40]. Overall, our results suggest a tactic of keeping still in a concealed spot to avoid detection, and fleeing only when very close to the perceived threat. This reliance on inconspicuousness might be efficient enough to avoid most detections by hunters, in the dark and often dense undergrowth of the rainforest.

Nevertheless, we acknowledge the limitations of our simulation exercise and do not claim the above estimates have any biological realism. Instead they should be taken for their pedagogical value only. Our estimate of blue duiker density at Lomako from the 1D distance sampling methodology was one order of magnitude larger than previous estimates elsewhere [17, 18, 41, 42]. This suggests that previous studies had even lower detection probability and stronger behavioural responses than us (Table 2). We would need to follow up on our surveys to confirm that this is effectively the case, i.e., actually implement the 2D method in the field. We also recommend that future users consider more complex behavioural response functions than we did, starting with the “sigmoid with intercept” function (“pi.sigmoI” in S1 Appendix). We did not find this function to yield qualitatively better results than the sigmoid in our simulations, but in a real case this model should be considered among the plausible ones.

In addition, because we recorded both dung piles (by day) and direct observations (by night), we could estimate the conversion rate of dung pile density to population density. The estimate of this conversion rate was sensitive to the behavioural response, meaning that the 2D framework should be very useful for this purpose too. Previous authors derived the conversion rate indirectly using the defecation rate of animals, as recorded in captivity or during short periods in the wild, and/or the decay rate of dung piles until they disappeared in controlled conditions [43–47]. However, this approximation is not considered reliable because the defecation and decay rates vary over time, across individuals and habitats. The conversion rate must be estimated in the same context where it will be applied, not under controlled conditions. Our 2D distance sampling protocol makes this possible: we first estimate dung pile density and animal density separately, which opens the possibility to only monitor the dung piles for the rest of the season or in similar study areas. We recommend future authors publish as much metadata as possible along with their conversion rate estimates, in order for the published conversion rates to be used by others who work in the same context, and for a meta-analysis to uncover why and how the conversion rate varies.

As a side note, the small size of the dung pellets of blue duikers is what makes it possible to assign the dung piles to that species; similar assignments are more difficult in larger species of duikers. Our field protocol also entailed a somewhat subjective assessment of the freshness of the dung piles in order to record the most recent dung piles only. Instead, one may record all the dung piles along with a qualitative score of their state, and apply the principles described in the rich literature about how to account for the variation in dung pile age, the rate at which dung piles decay, and the number of defecation events per day [43–50].

What our study clearly demonstrates is that the bias caused by the improper treatment of the behavioural response of the animals to the transects or the observers may cause the estimates of population density to artificially vary by a factor 2 to 3. This can severely flaw the comparison of duiker abundance from one site to another, or from hunted locations to hunting-free areas.

### Implications for conservation

Many bushmeat species have been observed to persist much longer than expected from theoretical models (reviewed by [10]). This might be because the theoretical models were parameterized with estimates of population density that were biased (e.g., Table 4 in [10]). Indeed, the negative bias that we illustrated in this study increases with the behavioural response; and the behavioural response expectedly increases with the hunting pressure [27, 31]. Therefore, the effect of the hunting pressure on the population density would be over-estimated, probably severely. Using a more adequate sampling protocol and statistical approach, such as the ones we recommend in this study, could thereby benefit all the involved parties. Conservationists would be able to recommend less stringent reductions in hunting pressure, that are less likely to generate compliance issues among hunters. Hunters would find it easier to adopt the recommendations of conservationists and thereby obtain the sustainability benefits.

Another potentially important implication of our findings is that estimating the behavioural response to the transects would also document the surface of habitat effectively lost by duikers due to the presence of hunting trails. This information could be incorporated into models of habitat availability and human footprint. One could maybe use the response to the observers to represent the effect of heavily used tracks, and the response to the transects to represent the effect of smaller paths. In our case, we estimated that the effect of the transect was mostly non-existent beyond 5–8 meters from the transects, but that within that strip duikers were c.30% less abundant. We could theoretically use a map of all the transects and trails in the Lomako reserve to extrapolate the overall loss in duiker habitat. As a side note, some species might be attracted, not repelled, by the transects, because the transects represent an ecotone and a change in vegetation structure [15, 29, 51].

### Brief overview of the alternatives to distance sampling

Now that we have discussed the benefits but also the challenges of distance sampling for duikers in the rainforest, we are providing a brief overview of the alternative methodologies that could be used instead of transect-based distance sampling, for the same objective of monitoring duiker abundance. This should serve as an argument as to why we are advocating the 2D distance sampling methodology. Namely, the alternatives are in our opinion unlikely to perform better.

The N-mixture model [52] is a classical methodology that relies on repeated counts of the same population. In the case of duikers, this would entail walking the transects multiple times and recording the number of duikers per transect or per kilometre (not necessarily recording the distance). Barker et al. [53] however warn that the estimates from this model are unreliable when the number of transects is low (<20) or when the average detection probability is low (<20%; see also [54]). Duiker studies typically fall below these thresholds, suggesting that the N-mixture framework may be too ambitious for duiker studies.

The frequency of animal pictures taken by automatic “camera traps” has also been proposed as an index of abundance [55], including in a context very similar to ours [56, 57]. However, the camera-trapping approach may be jeopardized by camera failures and high maintenance costs in the rainforest environment, by poachers, and remains relatively imprecise in the absence of information about the individual identity of the photographed animals, and their local fine-scale microhabitat selection and daily range [40, 41, 55]. Interestingly, Howe et al. [57] used distance sampling on camera trapping data, by placing distance markers in the field of view of the cameras. They and others report a behavioural response to the cameras similar to the one we describe [56, 57]. This strongly suggests that the 2D methodology is necessary to apply distance sampling to camera trapping data. As a side note, for camera trapping data, the radial data format (radial distance plus observation angle) is more appropriate than the (x,y) data format that we used in the present study.

Another alternative is to quantify the home range size and home range overlaps using movement data, and in a second step compute the population density as the number of home ranges per unit of space [42, 58]. However, it can be challenging to catch and release a large number of GPS-fitted duikers, and, because duikers live in shady undergrowth and groom themselves quite vigorously, the GPS devices would need to be battery-powered (vs. solar powered). Given the small weight of duikers, and therefore the small allowable weight of the battery, the tracking duration would therefore be severely limited, which is a known factor of bias in home range studies [59].

### Technical recommendations for future distance sampling studies

We strongly recommend recording both the forward and perpendicular distances, or alternatively the radial distance and the observation angle. Currently, in studies of rainforest mammals, researchers record only the perpendicular distance, or only the radial distance [17, 18, 56, 57]. We acknowledge that we did not actually test the feasibility of that recommendation in the field, but recording the forward distance does not seem to incur much more effort, nor require more skills, than recording the perpendicular distance, especially if using a hand-held GPS in transect-based distance sampling.

We recommend a close inspection of perpendicular distance sampling data collected from forest mammals to assess whether the patterns that we describe in Fig 1 occur or not. Importantly, the diagnosis may be flawed if binning the data at a too coarse level in the distance histograms. For forest-dwelling mammals, we recommend bins of 1 or 2 meters, not more, to limit the risk of overlooking the behavioural response.

Wherever possible, we also recommend having two observers perform the same protocol, keep separate logs, and then compare observations within a formal algorithm [54, 60, 61].

For the most challenging cases, when the behavioural response is extreme, we further recommend a replicated sampling approach, *i*.*e*. surveying the same transects multiple times per season. These last two recommendations aim at allowing a switch to index-based approaches instead of distance analysis (see below for more discussion of index-based approaches).

### Perspective into the use of other indicators of ecological change

When the populations of large herbivores such as duikers fluctuate around the carrying capacity, which itself may vary through time, various density-dependent processes are triggered or suppressed [12]. Typically, when the population is above carrying capacity, individuals and especially young individuals struggle to acquire resources and they become leaner and grow slower than when the population is below carrying capacity [13, 14, 62]. By contrast, when the population is below carrying capacity, either because the ecosystem has been more productive than usual or because hunting has reduced the population size too much, young animals should develop faster and grow larger. It is thus theoretically possible to use various biometric data collected from harvested individuals, such as the dressed (gutted) body mass or the hind leg length, as indicator of the status of the population-environment relationships [11, 14]. These data could be collected either locally by the hunters themselves, or at selling points by researchers. However, at least until the functioning of the population is completely understood, these indicators of body condition are best combined with an indicator of population abundance, with an indicator of food availability, and an indicator of the intensity of herbivory [11]. For duikers, the population abundance would be tracked through distance sampling as described in this article. The food resource to monitor would be fallen fruit on the forest floor. Lastly, changes in community composition can also be used as an ecological indicator. For example, it has been demonstrated several times that the ratio of blue duikers to larger species of duiker increases along gradients of hunting pressure [15, 63], probably under the combined influence of hunter selectivity and of the faster pace of life of the blue duiker relative to larger species. The change over time in the ratio of large to small species [8] or the ratio of long-lived to short-lived species [64] could therefore constitute an efficient indicator of the defaunation process.

## Supporting information

S1 Appendix Code appendix including the functions to specify our model and its variants, including some that accommodate the violation of the working hypotheses in this article, and the script to reproduce the simulations. (ZIP)
